# Exploration of the Interaction Between Bismuth-Containing Quadruple Anti-*H. pylori* Therapy and the Gut Microbiota

**DOI:** 10.3390/microorganisms14081749

**Published:** 2026-08-08

**Authors:** Xue Yang, Yan Pan, Cai-Ping Gao, Ying-Hui Zhang, Shan Du, Lu Chao, Chun-Li Huang, Shi-Yu Xiao, Zhou Zhou

**Affiliations:** Department of Gastroenterology, Sichuan Provincial People’s Hospital, School of Medicine, University of Electronic Science and Technology of China, Chengdu 610000, China; yangxue_2001@163.com (X.Y.);

**Keywords:** *Helicobacter pylori*, compound *Lactobacillus acidophilus*, bismuth-containing quadruple therapy, gut microbiota

## Abstract

*Helicobacter pylori* (*H. pylori*) eradication with bismuth-containing quadruple therapy (BQT) is effective but often accompanied by adverse gastrointestinal reactions and gut microbiota disturbances, while the impact of probiotic supplementation and the predictive value of the gut microbiota remain incompletely understood. In this single-center prospective randomized controlled trial, 109 *H. pylori*-positive patients were assigned to receive a 14-day BQT alone treatment or BQT combined with compound *Lactobacillus acidophilus* (LBQT). Fecal samples were collected before treatment, at the end of therapy, and four weeks after completion for 16S rDNA amplicon sequencing to assess microbiota dynamics. Although the addition of compound *L. acidophilus* did not significantly alter eradication rates, it markedly reduced the incidence of gastrointestinal adverse reactions, accelerated the recovery of gut microbial diversity and abundance, and delayed the overgrowth of potentially pathogenic bacteria, as well as increased short-chain fatty acid-producing bacteria. We further predicted the success of *H. pylori* eradication using ROC curves, and found that the combined baseline fecal microbiota markers (*o_Lactobacillales*, *o_Enterobacterales*, and *f_Enterobacteriaceae*) achieved an AUC of 0.849 at the start of treatment; at 4 weeks after eradication, the AUC of the microbiota combined with *o_Peptostreptococcales*, *c_Bacilli*, *o_Lactobacillales*, and *g_Streptococcus* predicted successful eradication up to 0.776. These findings indicate that compound *L. acidophilus* supplementation alleviates the side effects of BQT and promotes the restoration of gut microbiota homeostasis, and that gut microbiota signatures may serve as noninvasive predictors of treatment tolerance and therapeutic outcome.

## 1. Introduction

*H. pylori* infection is an infectious disease that is strongly associated with the development of peptic ulcers, gastric mucosa-associated lymphoma, and gastric cancer [1]. Eradication of *H. pylori* has become a primary preventive measure for gastric cancer [2]; In China, due to high incidence of gastric cancer, current guidelines explicitly recommend eradication therapy for all infected adults [3]. However, increasing antibiotic resistance has caused the eradication rate of standard triple therapy to fall below 80% [4]. Therefore, a 14-day quadruple regimen of a proton pump inhibitor combined with two antibiotics and bismuth is being recommended as a first-line eradication regimen by an increasing number of guidelines [5,6,7].

However, the use of multiple medications has led to an increase in adverse effects, including diarrhea, abdominal pain, nausea, abnormal taste, vomiting, and constipation [8]. At the same time, studies have shown that *H. pylori* eradication therapy leads to disturbances in the gut microbiota, and that the addition of probiotics to the anti-*H. pylori* regimen might alleviate the adverse effects and increase the success rate of eradication [9]. However, it is not clear whether the effect on the patient’s gut microbiota of adding probiotics to the regimen, nor is it clear whether there is any interaction between the anti-*H. pylori* therapy and the gut microbiota.

*Lactobacillus acidophilus* is a probiotic strain with multiple anti-*H. pylori* mechanisms. Previous studies have shown that it can break down sugars into lactic acid, thereby altering the gastric microenvironment and inhibiting *H. pylori* colonization and adhesion to the gastric mucosa [10]. Additionally, it secretes organic acids and bacteriocins, which exert a direct bactericidal effect against *H. pylori* [11].

In this study, by means of 16S rDNA, we explored the effects of quadruple therapy combined with compound *L. acidophilus* on the eradication of *H. pylori*, the gut microbiota, and whether the treatment causes adverse effects, as well as whether the gut microbiota could predict these adverse effects and the success of eradication.

## 2. Materials and Methods

### 2.1. Study Population

This was a single-center, prospective, interventional, randomized controlled clinical trial. Randomization was based on a computer-generated random number table. The inclusion criteria were (1) age 18–60 years; (2) a positive result for *H. pylori* infection according to at least one of the following methods: ① ^13^C or ^14^C urea breath test, ② fecal *H. pylori* antigen test, or ③ immunohistochemical staining of gastric biopsy specimens; and (3) asymptomatic patients willing to undergo first-line eradication therapy.

The exclusion criteria were (1) previous *H. pylori* eradication treatment; (2) a known allergy to any component of the study drugs; (3) other concomitant problems or conditions judged by the investigator to make the patient inappropriate for enrollment, including cardiopulmonary, hepatic, or renal diseases, tumors, inflammatory bowel disease, and a history of gastrointestinal surgery; (4) pregnancy; and (5) use within 3 months prior to enrollment of antibiotics, acid suppressants, bismuth preparations, probiotics, or other agents known to affect the gut microbiota.

This study aimed to elucidate the effects of probiotic supplementation on gut microbiota during quadruple therapy. As a real-world investigation, enrollment was limited by the financial burden borne by participants. Based on sample size estimations from comparable trials, a total of 109 patients were finally recruited [12]. The enrolled patients were randomly assigned to the BQT (esomeprazole 20 mg b.i.d, clarithromycin 500 mg b.i.d, amoxycillin 1000 mg b.i.d, colloidal bismuth pectin 200 mg b.i.d) and LBQT (esomeprazole 20 mg b.i.d, clarithromycin 500 mg b.i.d, amoxycillin 1000 mg b.i.d, colloidal bismuth pectin 200 mg b.i.d, compound *L. acidophilus* 1000 mg b.i.d) groups. Esomeprazole and colloidal bismuth pectin were taken orally 30–60 min before meals, amoxicillin and clarithromycin were taken orally 30–60 min after meals, and compound *L. acidophilus* was taken orally at noon, and at night before bedtime.

Compound *L. acidophilus* (Manufactured by Tonghua Jinma Pharmaceutical Co., Ltd., Jilin, China. Approval No.: H10940114) is a compound drug composed of the Chinese strain of *L. acidophilus*, the Japanese strain of *L. acidophilus*, *Streptococcus faecalis*, and *Bacillus subtilis*; each 1 g contains 10^7^ units of *L. acidophilus*.

Due to the nature of the intervention, patients and assessors were not blinded to group assignment. The study was registered with the China Clinical Trial Registry (ChiCTR2400084413) and filed in the Medical Research Registration and Filing Information System (MR-51-24-025116). It was approved by the Medical Ethics Committee of Sichuan Provincial People’s Hospital, and all participants provided written informed consent before enrollment.

### 2.2. Sample Collection and DNA Extraction

In this study, stool samples were collected for analysis of the bacterial microbiota at three time points: before treatment (V1), at the end of the 2-week anti-*H. pylori* regimen (V2), and 4 weeks after discontinuation of medication (V3). The breath test was performed 28 days after the withdrawal of all medication, including antibiotics and PPIs. A small spoon attached to the bottle cap was used to collect fresh fecal, which was placed in the stool tube, barcoded, numbered, and then frozen and stored in a −80 °C freezer. The DNA was extracted from the stool samples using the TGuide S96 Magnetic Stool DNA Kit (Tiangen Biotech (Beijing) Co., Ltd., Beijing, China) according to manufacturer’s instructions. The DNA concentration was measured using the Qubit dsDNA HS Assay Kit and Qubit 4.0 Fluorometer (Invitrogen, Thermo Fisher Scientific, Eugene, OR, USA).

### 2.3. 16S rRNA Full-Length Gene Sequencing and Bioinformatics Analysis

Full-length 16S rRNA genes were amplified using universal bacterial primers 27F (5′-AGRGTTYGATYMTGGCTCAG-3′) and 1492R (5′-RGYTACCTTGTTACGACTT-3′). Each 25 μL PCR reaction contained 12.5 μL 2× Taq Plus Master Mix (Vazyme, Nanjing, China), 2.5 μL of each primer (10 μM), and 10 ng template DNA. Thermal cycling conditions comprised initial denaturation at 95 °C for 4 min; 35 cycles of 95 °C for 15 s, 55 °C for 30 s, and 72 °C for 2 min; and a final extension at 72 °C for 7 min. Amplicons were verified by 2% agarose gel electrophoresis and purified using AMPure PB Beads (PacBio, Menlo Park, CA, USA). Sequencing libraries were prepared using the SMRTbell Express Template Prep Kit 2.0 (PacBio) and sequenced on a PacBio Sequel II platform (Biomarker Technologies, Beijing, China) in circular consensus sequencing (CCS) mode. Raw subreads were processed with SMRT Link v10.1. CCS reads were generated by consolidating multiple passes of the same molecule and filtered based on predicted accuracy ≥ 99% and length between 1200 and 1650 bp. High-quality CCS reads were denoised and clustered into amplicon sequence variants (ASVs) using the DADA2 algorithm implemented in QIIME2 (v2022.2). Chimeric sequences were removed using the consensus method. Taxonomic assignment was performed with a Naïve Bayes classifier trained on the SILVA reference database (release 138).

Alpha diversity metrics (using the Chao1, ACE, Shannon, and Simpson indices) were calculated using the QIIME2 diversity plugin. The beta diversity was assessed using the Bray–Curtis dissimilarity and visualized by principal coordinates analysis (PCoA). Differentially abundant taxa between groups were identified using the Linear Discriminant Analysis Effect Size (LEfSe) pipeline, with significance defined as an LDA score > 2.0 and a Kruskal–Wallis *p* < 0.05. All bioinformatic workflows and visualizations were conducted on the BMK Cloud platform (Biomarker Technologies, Beijing, China).

### 2.4. Statistical Analyses

Clinical and microbial data were analyzed using SPSS version 21.0 (IBM Corporation, Armonk, NY, USA). Quantitative data were reported as mean ± standard deviation, while qualitative variables were expressed as numbers or percentages. Normality was assessed using the Shapiro–Wilk test. Between-group comparisons of normally distributed continuous variables were performed using Student’s *t*-test or one-way ANOVA, whereas non-normally distributed data were analyzed using the Mann–Whitney U test or Kruskal–Wallis H test. Paired comparisons were conducted using the Wilcoxon signed-rank test. Categorical variables were compared using the chi-square or Fisher’s exact test, as appropriate. *H. pylori* eradication rates were evaluated by both intention-to-treat (ITT) and per-protocol (PP) analyses.

For microbiome data, beta diversity differences were formally tested using permutational multivariate analysis of variance (PERMANOVA) based on the Bray–Curtis dissimilarity. To address multiple comparisons inherent in high-dimensional microbiome data, all univariate *p*-values from taxonomic and diversity analyses were corrected using the Benjamini–Hochberg false discovery rate (FDR) procedure. An FDR-adjusted two-tailed *p* < 0.05 was considered statistically significant. Receiver operating characteristic (ROC) curves were constructed to evaluate the discriminatory capacity of selected microbial biomarkers, and the area under the curve (AUC) was calculated.

## 3. Results

### 3.1. Characteristics of the Study Population

A total of 109 patients with *H. pylori* infection were finally enrolled in this study. The enrolled patients were randomly assigned to the BQT and LBQT groups. Compliance was defined as complete adherence to the assigned treatment regimen (all doses taken as scheduled) and the successful collection of stool samples at all the required time points. In our preliminary clinical exploration, we observed that in real-world settings, the ratio of patients opting for anti-*H. pylori* therapy without probiotic supplementation versus those with probiotic supplementation was approximately 4:3. This disparity may be related to treatment costs and patient preferences. Consequently, we adopted a 4:3 grouping ratio throughout the study.

Eventually, a total of 62 patients were enrolled in the BQT group, four were withdrawn, one was lost without review, and 173 stool samples were collected. In the LBQT group, 47 cases were enrolled, three cases withdrew, two cases were lost without review, and 124 stool samples were collected.

There were no statistically significant differences between the two groups in terms of gender, age, or eradication rate. The ITT eradication rate in the BQT group was 79.03%, and the PP eradication rate was 85.96%; the ITT eradication rate in the LBQT group was 78.72%, and the PP eradication rate in the LBQT group was 88.10%. No statistically significant differences were observed in either the ITT or PP eradication rates. This indicates that the addition of compound *L. acidophilus* had no effect on the eradication rate of *H. pylori.* However, there was a significant difference in the occurrence of adverse reactions between the two groups, and this study mainly included patients’ gastrointestinal adverse reactions, including abdominal pain, diarrhea, bloating, and constipation. Adverse events were summarized as a composite measure; all events were mild and transient, and overlapping symptoms in some patients precluded reliable classification by individual symptom type. This suggested to us that the addition of probiotics could significantly reduce the adverse gastrointestinal effects of *H. pylori* eradication (Table 1).

### 3.2. Effect of Quadruple Eradication Therapy on Fecal Microbiota

**Figure 1:**
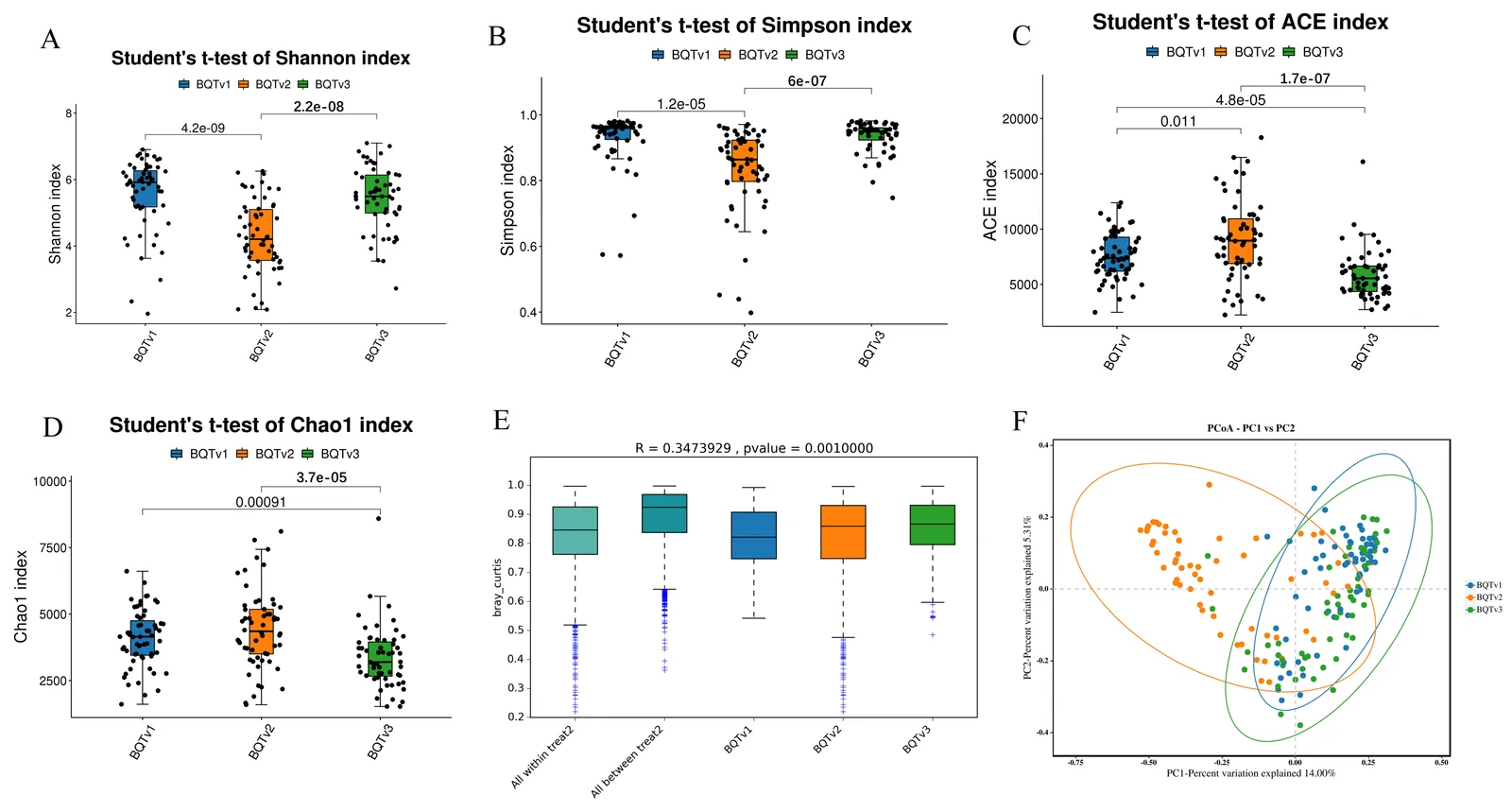
Alpha and beta diversity analysis of fecal microbiota following quadruple eradication therapy. Alpha diversity indices were estimated using the values of the observed Shannon index (**A**), Simpson index (**B**), ACE (**C**), and Chao index (**D**); beta diversity assessment was obtained by Anosim analysis (**E**) and PCoA (**F**). In Anosim analysis, R values closer to 1 indicate that between-group differences are greater than within-group differences, while smaller R values indicate that there are no significant between- and within-group differences, and *p* values below 0.05 indicate that the test had a high level of confidence.

**Figure 2:**
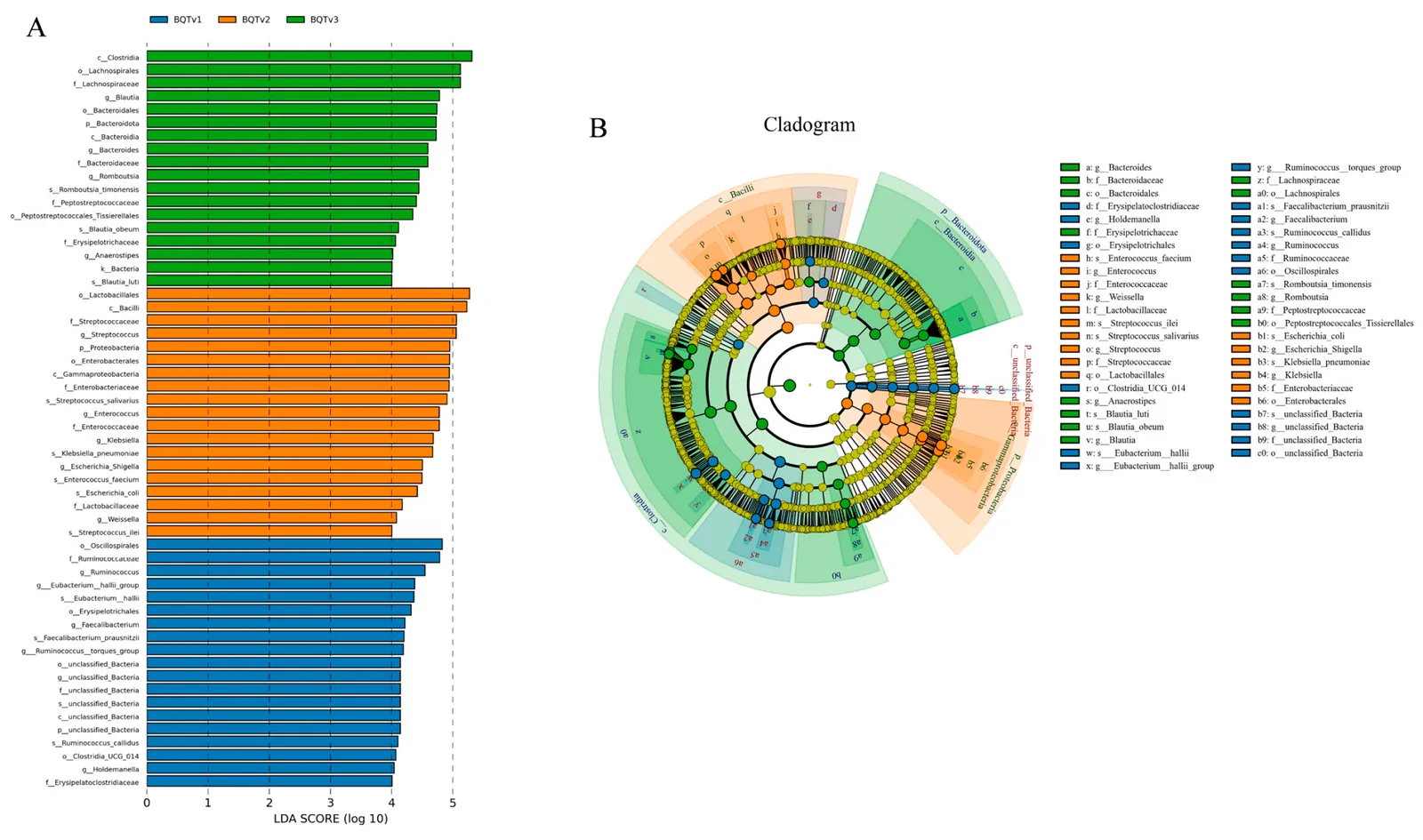
Differences in fecal microbiota over time for BQT group treatments. (**A**): Differences in microbiota over time through LEfSe analysis; (**B**): differences in microbiota over time represented as a branching evolutionary map.

We measured species richness using the Chao1 (Figure 1D) and Ace indices (Figure 1C), and species diversity using the Shannon (Figure 1A) and Simpson indices (Figure 1B). It was found that quadruple eradication therapy reduced the diversity of the microbiota at the end of treatment (V2) and restored baseline levels 4 weeks after discontinuation (V3), whereas species richness did not return to baseline levels after 4 weeks. This suggests that the effect of quadruple therapy on the diversity of the gut microbiota was transient, whereas the effect on species richness was of a longer duration and should be observed over a longer time period. In addition, beta diversity analysis identified significant differences in fecal bacteria before and after quadruple therapy treatment (Figure 1E,F). LEfSe analysis further revealed a significant increase in the relative abundance of pathogenic bacteria, for example, *g-Escherichia_Shigella* and *s-Escherichia_coli*, at V2 (Figure 2A,B), which may create a window of opportunity for gut microbiota disruption and increase the risk of disease.

### 3.3. Effect on Fecal Microbiota of Adding Compound L. acidophilus to Quadruple Therapy

**Figure 3:**
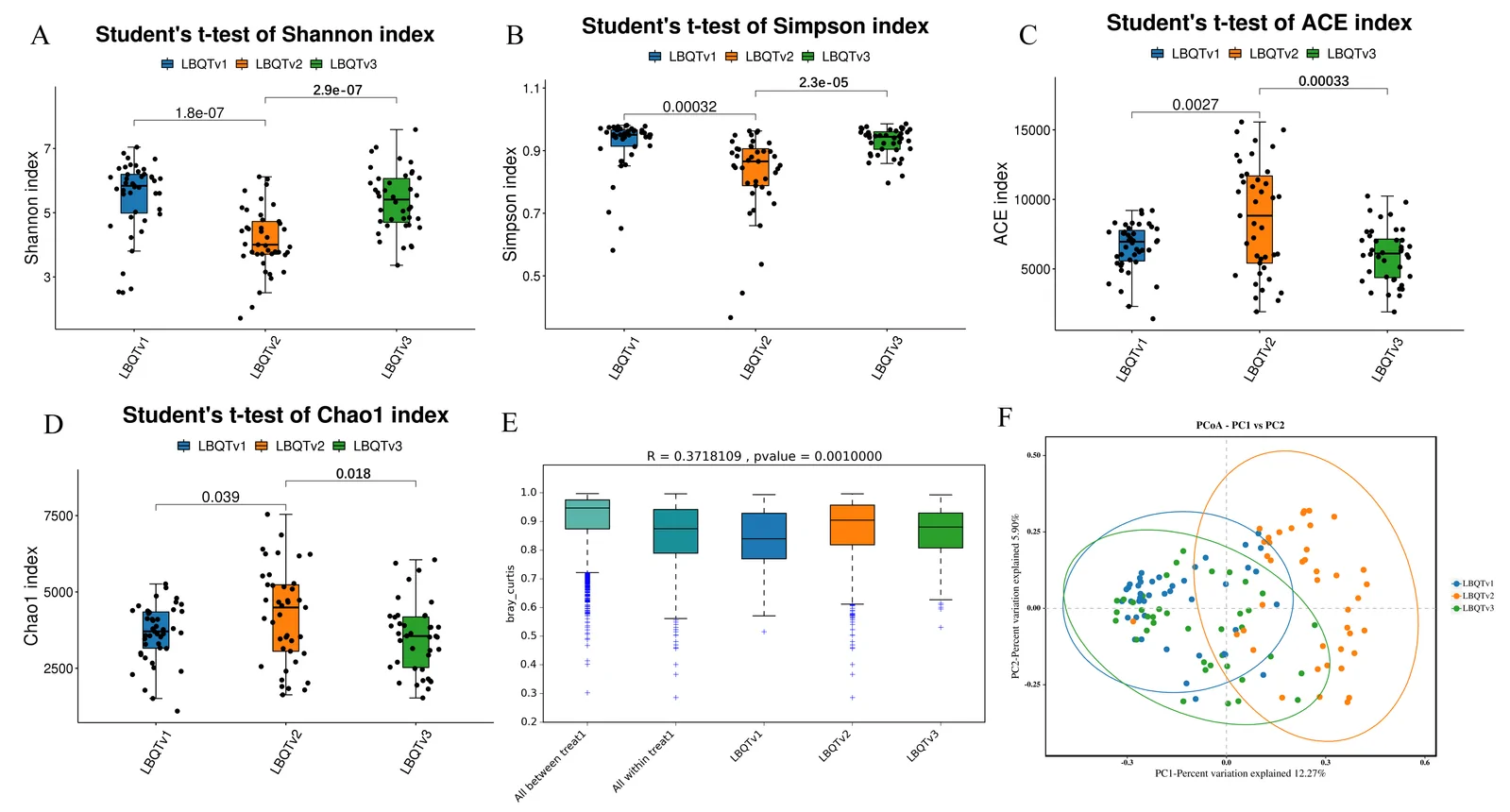
Alpha and beta diversity analysis of fecal microbiota under quadruple eradication therapy with the addition of compound *L. acidophilus*. Alpha diversity indices were estimated using the value of the observed Shannon index (**A**), Simpson index (**B**), ACE (**C**), and Chao index (**D**); beta diversity assessment was obtained by Anosim analysis (**E**) and PCoA (**F**).

**Figure 4:**
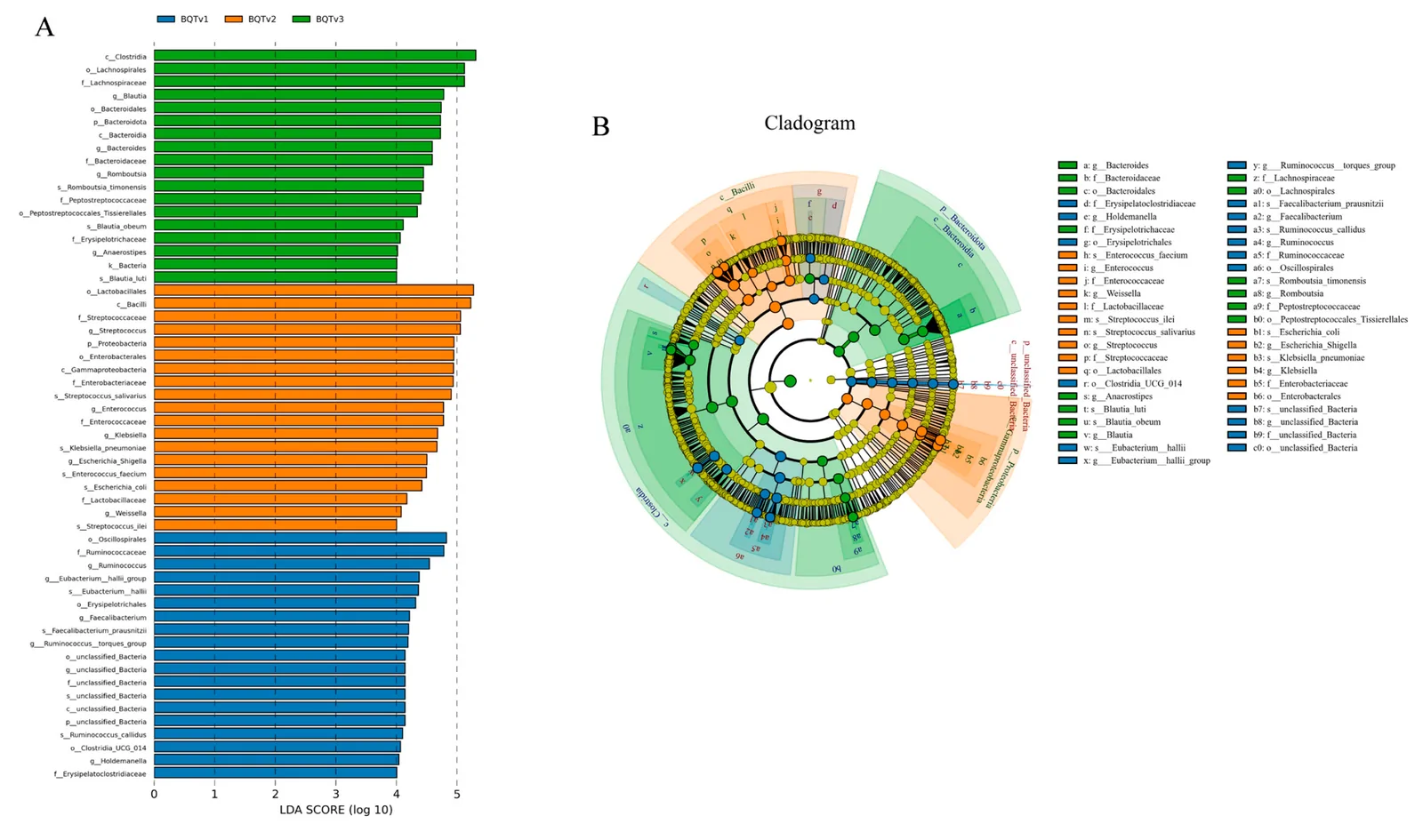
Differences in fecal microbiota over time for LBQT group treatments. (**A**): Differences in microbiota over time through LEfSe analysis; (**B**): differences in microbiota at over time represented as a branching evolutionary map.

After the addition of compound *L. acidophilus* to quadruple therapy, we observed a decrease in the diversity of the fecal microbiota at 2 weeks (V2) and a return to baseline levels at 4 weeks (V3) (Figure 3A,B). Meanwhile, fecal microbiota abundance increased slightly at 2 weeks (V2) and returned to pre-treatment baseline levels at 4 weeks (V3) (Figure 3C,D), suggesting that the addition of compound *L. acidophilus* favored gut microbiota abundance. Moreover, the addition of compound *L. acidophilus* resulted in differences in fecal microbiota beta diversity at the three time points (Figure 3E,F). The increase in the relative abundance of pathogenic bacteria, such as *g_scherichia_Shigella* and *s_Escherichia_coli*, appeared at the discontinuation of medication at 4 weeks (V3) (Figure 4A,B). The addition of compound *L. acidophilus* delayed the peak time of the occurrence of some pathogenic bacteria.

### 3.4. Differences in Fecal Microbiota Between the Two Groups at Different Time Points

**Figure 5:**
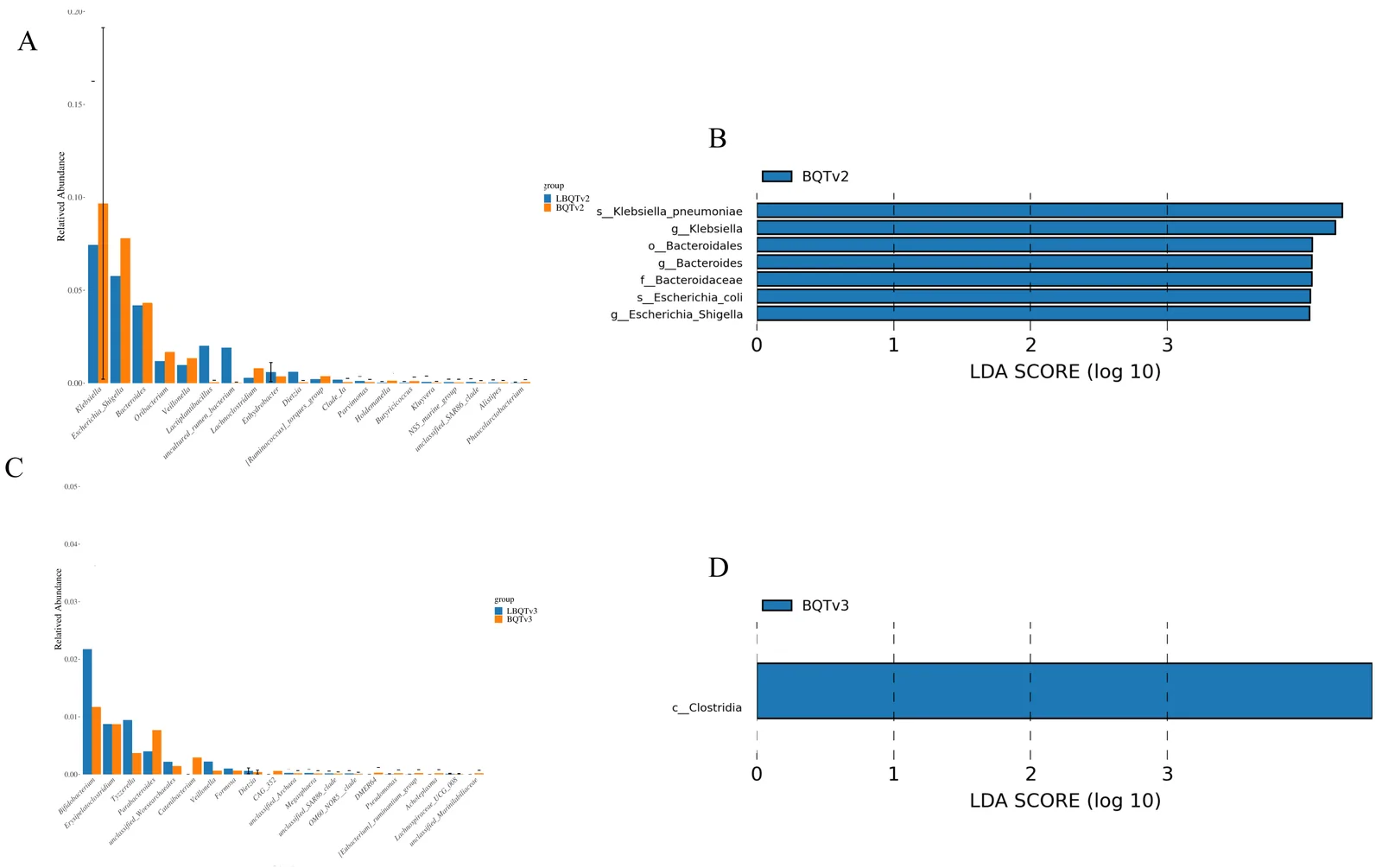
Differences in fecal microbiota between the two groups over time. (**A**): Differences in fecal microbiota between the two groups were analyzed using the Wilcox test at the V2 time point; (**B**): differences in fecal microbiota between the two groups were analyzed using LEfSe analysis at the V2 time point; (**C**): differences in fecal microbiota between the two groups were analyzed using the Wilcox test at the V3 time point; (**D**): differences in fecal microbiota between the two groups were analyzed using LEfSe analysis at the V3 time point.

We compared the differences in fecal microbiota between the two groups at the three time points after the addition of compound *L. acidophilus*. We found that the relative abundance of pathogenic *g_Klebsiella*, *s_Klebsiella_pneumoniae*, and *g_Escherichia_shigella* in the BQT group was higher than that in the LBQT group at 2 weeks (V2) after eradication, which increased the risk of pathogenicity (Figure 5A,B). At 4 weeks post treatment (V3), the LBQT group exhibited a higher relative abundance of taxa reported to be involved in short-chain fatty acid (SCFA) production, such as *Bifidobacterium* and *Veillonella*, whereas the abundance of *Clostridia* was greater in the BQT group (Figure 5C,D). Notably, *Bifidobacterium* [13] and *Veillonella* [14] have been widely implicated in SCFA production; however, as fecal SCFA levels were not quantified herein, we speculate that there may be SCFA differences between the two groups.

### 3.5. Fecal Microbiota Predicted Adverse Events in Quadruple Therapy

**Figure 6:**
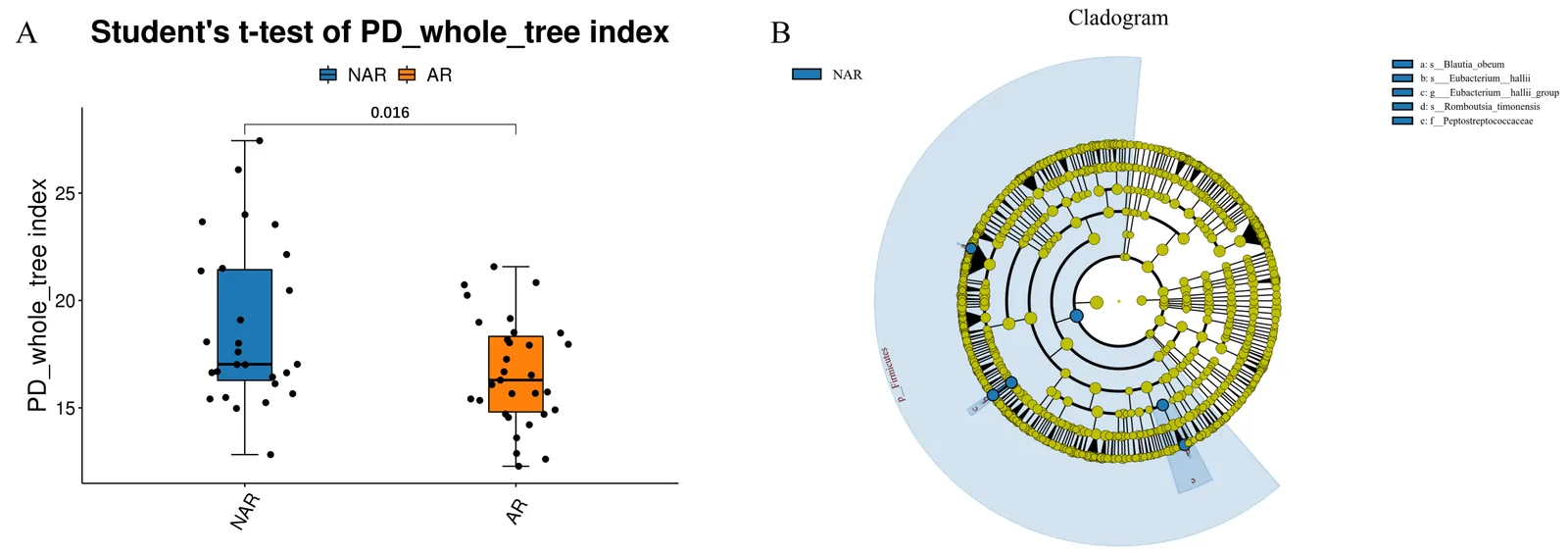
Fecal microbiota predicted adverse events in quadruple therapy. (**A**): Differences in bacterial diversity between the two groups at the baseline level were analyzed using the PD whole-tree index. (**B**): Branching evolutionary map demonstrating the differences in fecal microbiota between the two groups at time point V3. Circles radiating from inside to outside of the evolutionary branching diagram represented taxonomic levels from phylum to species. Each small circle at a different taxonomic level represents a classification at that level, with species that were not significantly different in yellow and species that were significantly different in blue.

Throughout the 2-week quadruple therapy and subsequent 4-week follow-up, all intestinal adverse reactions, including abdominal pain, diarrhea, bloating, and constipation, were mild and self-limiting, with no severe events requiring clinical intervention. We compared patients with adverse reactions in the BQT group with patients without adverse reactions using PD_whole_tree, and found that baseline bacterial microbiota diversity was higher in those who experienced no intestinal adverse reactions than in those did experience them (Figure 6A). In addition, at observation point V3, it was found that those without intestinal adverse reactions in the BQT group had an increase in beneficial bacteria such as *p_Firmicutes*, *s_Romboutsia_timonensis*, *f_Peptostreptococcaceae*, *g_Eubacterium_hallii_group*, *s_Eubacterium_hallii*, and *Blautia_obeum* (Figure 6B), several of which have been previously linked to SCFA metabolism, especially butyrate production and cross-feeding pathways [15]. These findings suggest a potential association between baseline microbial composition and treatment tolerance; however, as fecal SCFA concentrations were not quantified in this study, the role of SCFAs in mitigating anti-*H. pylori*-related adverse reactions remains a matter for further investigation.

### 3.6. Fecal Microbiota Predicted Quadruple H. pylori Eradication Therapy Success

**Figure 7:**
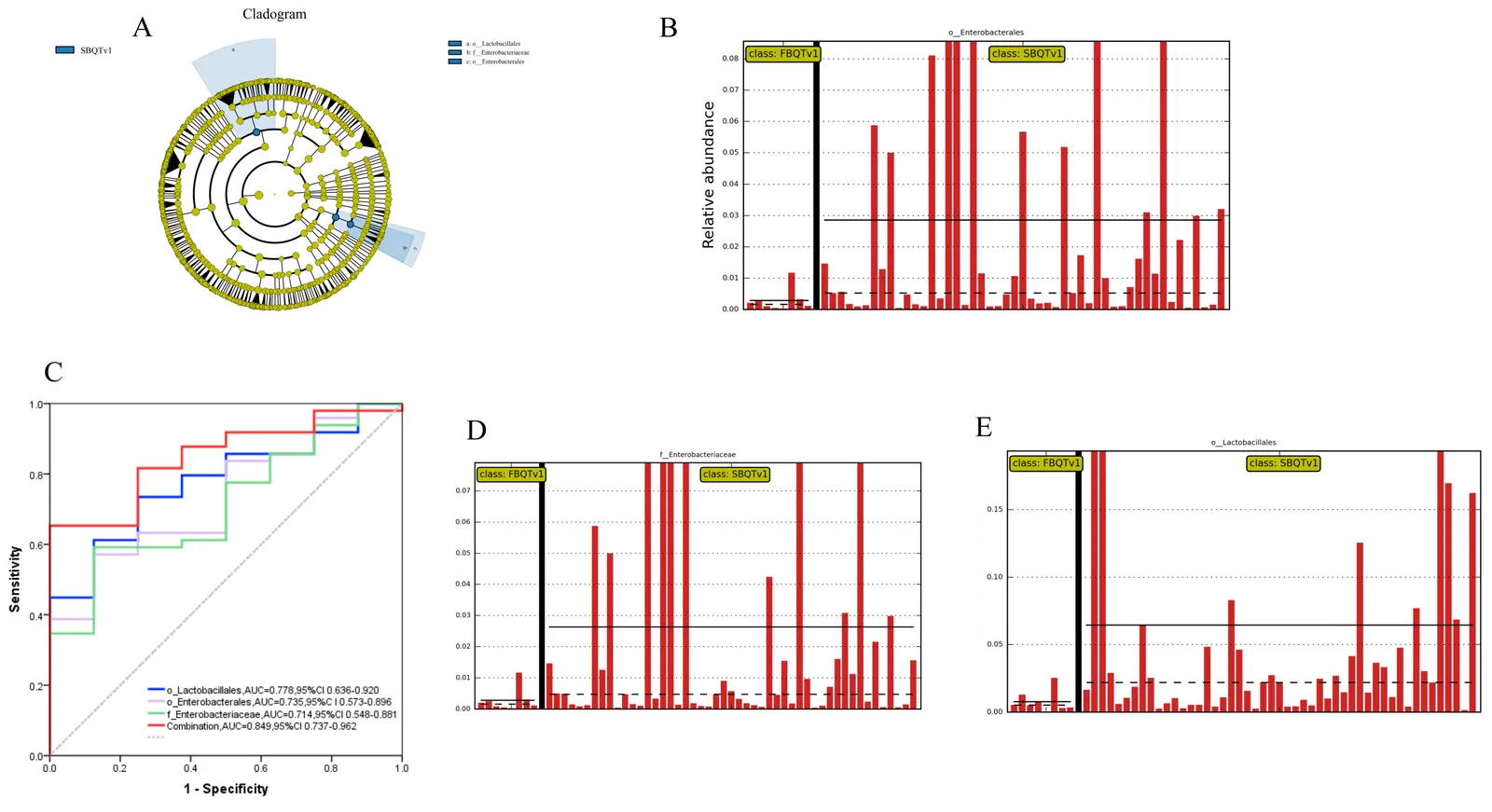
Baseline fecal microbiota predicted the success of quadruple *H. pylori* eradication therapy. (**A**): Evolutionary branching plots demonstrated differences in bacterial populations between the eradication success and eradication failure groups at baseline; (**B**): LEfSe analysis of the relative abundance distribution of *o_Lactobacillales* in both groups; (**C**): predicting eradication success by AUCs at the V1 time point; (**D**): LEfSe analysis of the relative abundance distribution of *f_Enterobacteriaceae* in both groups; (**E**): LEfSe analysis of the relative abundance distribution of *o_Enterobacterales* in both groups. In (**B**), (**D**) and (**E**), the mean and median relative abundance values in each subgroup are identified by solid and dashed lines, respectively.

**Figure 8:**
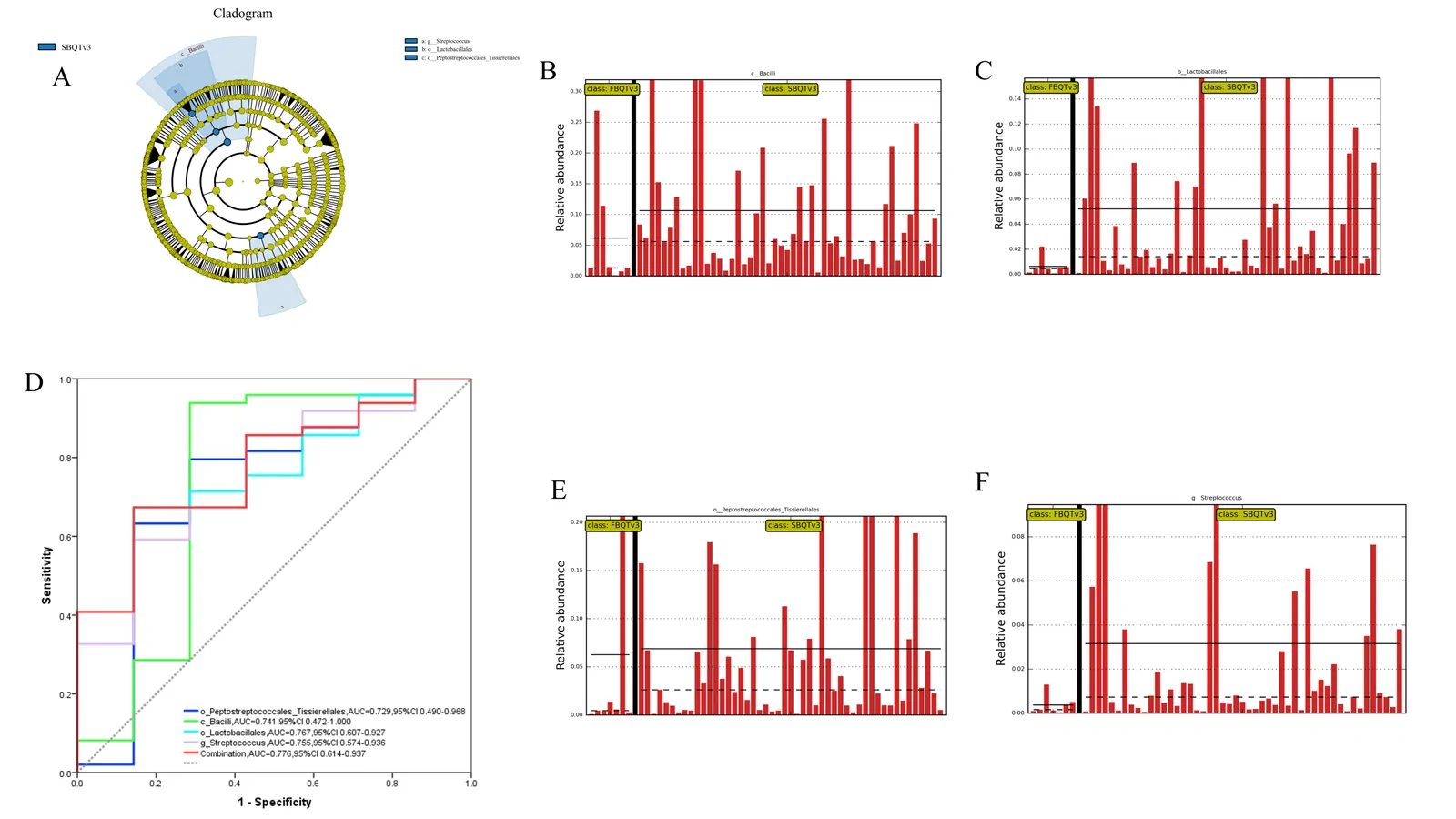
Fecal microbiota at 4 weeks post treatment predicted successful *H. pylori* eradication following quadruple therapy. (**A**): Evolutionary branching plots demonstrate differences in bacterial populations between the eradication success and eradication failure groups 4 weeks after sterilization; (**B**): LEfSe analysis of the relative abundance distribution of *c_Bacilli* in both groups; (**C**): LEfSe analysis of the relative abundance distribution of *o_Lactobacillales* in both groups; (**D**): predicting eradication success by AUCs at the V3 time point; (**E**): LEfSe analysis of the relative abundance distribution of *o_Peptostrepto*coccales in both groups; (**F**): LEfSe analysis of the relative abundance distribution of *g_Streptococcus* in both groups. In (**B**), (**C**), (**E**) and (**F**), the mean and median relative abundance values in each subgroup are identified by solid and dashed lines, respectively.

Some previous studies have found that the addition of probiotics might improve the effect of anti-*H. pylori* therapy [16], but other studies do not support this view [17]. In our study, the addition of compound *L. acidophilus* was not found to significantly improve *H. pylori* eradication rates, but fecal microbiota did correlate with the success of *H. pylori* eradication (Figure 7A and Figure 8A). At the V1 observation point, eradication successes resulted in higher relative abundances of baseline *o_Lactobacillales* (Figure 7B), *o_Enterobacterales* (Figure 7E), and *f_Enterobacteriaceae* (Figure 7D). At observation point V3, the relative abundances of *o_Peptostreptococcales* (Figure 8E), *c_Bacilli* (Figure 8B), *o_Lactobacillales* (Figure 8C), and *g_Streptococcus* (Figure 8F) were higher. At the same time, much of the increase in the fecal microbiota in the successful eradication group was attributable to bacteria taxonomically affiliated with known SCFA-producing groups, such as *o_Lactobacillales*, *o_Peptostreptococcales*, and *g_Streptococcus* [18].

We further predicted the success of *H. pylori* eradication using AUCs and found that the AUC of the baseline fecal microbiota combined with *o_Lactobacillales*, *o_Enterobacterales*, and *f_Enterobacteriaceae* predicted successful eradication up to 0.849 (95% CI 0.737-0.962) (Figure 7C), and the AUC of microbiota combined with *o_Peptostreptococcales*, *c_Bacilli*, *o_Lactobacillales*, and *g_Streptococcus* predicted successful eradication up to 0.776 (95% CI 0.614-0.937) at 4 weeks (V3) (Figure 8D). In conclusion, we might be able to predict the anti-*H. pylori* success by analyzing the fecal microbiota of patients; however, in future research, we would also need to expand the sample size.

## 4. Discussion

*H. pylori* is a transmissible Gram-negative bacterium that can persistently colonize the stomach. Long-term infection can lead to inflammatory damage to the gastric mucosa, atrophy of gastric intrinsic glands, and intestinal metaplasia [19]. In 1994, the International Agency for Research on Cancer (IARC) classified *H. pylori* as a class I carcinogen, and it is the most important risk factor for the prevention of gastric cancer, eradication of *H. pylori* is the primary preventive measure for gastric cancer, which is of great significance [20].

At present, various new treatment programs, including sequential therapy [21], concomitant therapy [22], and dual therapy [23], have been introduced globally. In general, quadruple combination therapy [7], which includes PPI, two antibiotics and bismuth, is the first-line treatment program for *H. pylori* eradication. However, there are some problems with these treatment programs. First, the antibiotics used in the regimen disrupt the normal microbiota of the digestive tract and cause microecological disturbances [24], which may lead to side effects such as nausea, vomiting, loss of appetite, and diarrhea. Secondly, antibiotic resistance is on the rise, and drug-resistant *H. pylori* passes on its drug-resistant genes through horizontal gene transfer [25], which will pose a serious challenge to future treatments for *H. pylori*. In addition, treatment with bismuth and PPI can also cause damage to patients’ gut microbiota [26,27], which is why many scholars have been exploring new therapies with superior eradication rates and controlled side effects.

Among these routes of exploration, the addition of probiotics to anti-*H. pylori* regimens has attracted great interest from researchers [28]. First, probiotics may play a protective role in terms of the gut microbiota disruption caused by anti-*H. pylori* therapy and may reduce gastrointestinal side effects [29]. Indeed, some probiotics can reduce *H. pylori* colonization and even have anti-*H. pylori* effects themselves [30]. Presently, some clinical studies have found that adding probiotics such as *Bifidobacterium* [31], *Streptococcus* [32], and *Pediococcus acidilactici* [33] can improve the bactericidal rate of the treatment and reduce the adverse reactions [27], but this approach is not currently without its problems. For example, there is substantial heterogeneity in terms of bacterial strains, dosages, and administration regimens across existing studies; therefore, the role of probiotics in *H. pylori* eradication therapy remains controversial [34].

In this study, we investigated the effects on *H. pylori* eradication rates, gut microbiota composition, and treatment-related adverse events of quadruple therapy supplemented with compound *L. acidophilus*. Furthermore, we explored the predictive potential of gut microbiota profiles for both therapeutic efficacy and adverse outcomes.

The compound *L*. *acidophilus* preparation used in this study is widely employed in clinical practice, ensuring both the generalizability and reproducibility of our findings. This formulation comprises *L. acidophilus* (Chinese strain), *L. acidophilus* (Japanese strain), *Enterococcus faecalis*, and *Bacillus subtilis*. With a high concentration of *L. acidophilus* reaching up to 10^7^CFU/g, this probiotic can decompose sugar into lactic acid, destroying the alkaline environment needed for *H. pylori* growth and proliferation, preventing *H. pylori* colonization and adhesion, and exerting an anti-*H. pylori* effect [35]; meanwhile, it can improve the phagocytosis function of mononuclear macrophages and the activity of natural killer cells, regulate the immunity function, and prevent invasion by *H. pylori* [36]. Previous studies have found that *L. acidophilus* could inhibit the growth of *H. pylori* isolates in vitro and had anti-*H. pylori* effects [30]. Additionally, *L. acidophilus* represents an effective therapeutic option for antibiotic-associated diarrhea.

The results show that, although there were no statistically significant differences between the two groups in terms of ITT, PP, and compliance, the PP eradication rate was higher in the BQT combined with compound *L. acidophilus* (LBQT) group than in the bismuth-containing quadruple therapy (BQT) group, while ITT and compliance were higher in the BQT group, which may be related to differences in the medication regimen. However, the eradication rates for all eradication regimens were below 90%, which might be related to our choice of clarithromycin as the antibiotic component of the treatment. The 2024 ACG guidelines recommend that regimens containing clarithromycin should be used only after determining antibiotic susceptibility. Currently, however, due to drug availability and cost considerations, clarithromycin remains our commonly used first-line treatment regimen in clinical practice. Additionally, the incidence of gastrointestinal adverse reactions was significantly reduced in the LBQT group, indicating that the addition of probiotics could significantly reduce adverse reactions in patients.

In our study, we found that anti-*H. pylori* treatment decreased the abundance and diversity of the microbiota, and the diversity of the microbiota returned to pre-treatment levels after 4 weeks of drug discontinuation, while the abundance of the microbiota did not return to baseline levels in that timeframe. Although the addition of compound *L. acidophilus* to the anti-*H. pylori* treatment also showed a decrease in the abundance and diversity of the microbiota, both treatment groups were able to return to baseline levels 4 weeks after discontinuation, suggesting that compound *L. acidophilus* was able to restore gut microbiota homeostasis in 4 weeks. In addition, we found that the emergence of certain intestinal pathogens, such as *Shigella* and *Escherichia coli*, was delayed after the addition of compounded *L. acidophilus*. This might provide time to intervene in opportunistic infections that arise during anti-*H. pylori* therapy.

We further analyzed the differences in fecal microbiota between the two groups at different time points, and we found that, at the V3 time point, the relative abundance of taxa known to be associated with SCFA production, such as *Bifidobacterium breve* and *Veillonella* spp., was higher in the LBQT group than that in the BQT group, and the relative abundance of *Clostridia* spp. in the BQT group was higher than that in the LBQT group. These compositional differences suggested a potential link between probiotic supplementation and an improved intestinal environment, although direct evidence of SCFA production requires further biochemical confirmation.

Previous studies, in addition to our study, have found that anti-*H. pylori* treatment had a significant effect on the gut microbiota, but whether the differences were suggestive of the development of adverse reactions in anti-*H. pylori* patients has not previously been studied. We detected differences in community diversity using PD_whole_tree and found that in the BQT group the baseline microbiota diversity of those without intestinal adverse reactions was higher than that those with intestinal adverse reactions. This suggests that the fecal microbiota was predictive of the occurrence of patients’ adverse reactions.

We further analyzed whether fecal microbiota could be predictive of anti-*H. pylori* treatment success. We found that the relative abundance of taxa potentially involved in SCFA production was associated with eradication success at time points V1 and V3, and that the AUCs for eradication success prediction after microbiota combination were 0.849 and 0.766 at time points V1 and V3, respectively, suggesting that there was a reciprocal relationship between gut microbiota composition and anti-*H. pylori* therapy. However, given the exploratory nature of this single-center study and the limited sample size, these predictive models require validation in larger independent cohorts before clinical application. This also suggests that, in future, it might be possible to reduce adverse reactions in eradication therapy and increase the success rate of anti-*H. pylori* treatment by increasing the diversity of the baseline microbiota, particularly by enriching taxa that are known to be associated with SCFA production.

Nevertheless, this study is subject to certain limitations. First, this was a single-center study, and the patients were not double-blinded, which might affect their assessment of adverse effects. Second, this study collected fecal microbiota but did not collect intestinal mucosal specimens, which might have affected the accuracy of the microbiota analysis. Third, the 4:3 allocation ratio between the two cohorts might introduce statistical bias. Furthermore, as a real-world observational study, the sample size was estimated based on previous studies, which remains modest and consequently constrains the definitiveness of our clinical inferences. Fourth, while we observed that SCFAs may be associated with reduced anti-*H. pylori* therapy side effects and improved treatment success rates, we were not able to measure concentrations in the gut and fecal content. In addition, this study used compound *L. acidophilus*, and did not analyze the effect of a single-bacteria probiotic.

This study provides significantly more information and evidence supporting changes to gut microbiota by compound *L. acidophilus* combined with quadruple therapy, and explores for the first time the predictive value of the gut microbiota regarding therapeutic efficacy and adverse reactions. At the same time, altering the patient’s baseline microbiota might reduce the side effects of anti-*H. pylori* treatment and improve gut microbiota composition. Future studies with expanded sample sizes are necessary to validate these preliminary results and facilitate their eventual integration into clinical practice.

## 5. Conclusions

The addition of compound *L. acidophilus* significantly reduced patients’ adverse reactions and also improved their gut microbiota composition. Meanwhile, fecal microbiota proved to be a predictor of adverse reactions and eradication success. Therefore, a reciprocal relationship between anti-*H. pylori* treatment and the gut microbiota is evident, and it is clinically important to optimize anti-*H. pylori* treatment regimens based on the gut microbiota.

## Figures and Tables

**Table 1:** Characteristics of study participants.

Variable	BQT (n = 62)	LBQT (n = 47)	*p*-Value	95% Cl
Age (year)	40.81 ± 9.73	38.81 ± 10.38	0.308	1.954 (−1.873, 5.877)
Sex (male/female)	23/39	18/29	0.898	0.950 (0.435, 2.077)
Compliance	57/62	42/47	0.645	1.357 (0.369, 4.990)
ITT (%)	79.03	78.72	0.969	1.019 (0.403, 2.578)
PP (%)	85.96	88.10	0.756	0.828 (0.250, 2.737)
Adverse reactions	30/62	6/47	0.000	6.406 (2.378, 17.258)

BQT: bismuth-containing quadruple anti-*H. pylori* therapy; LBQT: quadruple regimen with the addition of compound *L. acidophilus*; ITT: intentionality analysis; PP: per-protocol analysis.

## Data Availability

The original contributions presented in this study are included in the article. Further inquiries can be directed to the corresponding author.

## Abbreviations

The following abbreviations are used in this manuscript:

BQT	bismuth-containing quadruple therapy
LBQT	BQT combined with compound *L. acidophilus*
*H. pylori*	*Helicobacter pylori*
LEfSe	The linear discriminant analysis
ITT	intention-to-treat
PP	per-protocol
SCFA	short-chain fatty acid
